# How Can Static and Oscillating Electric Fields Serve in Decomposing Alzheimer’s and Other Senile Plaques?

**DOI:** 10.1021/jacs.2c12305

**Published:** 2023-02-03

**Authors:** Surajit Kalita, Hagai Bergman, Kshatresh Dutta Dubey, Sason Shaik

**Affiliations:** † Institute of Chemistry, The Hebrew University of Jerusalem, Edmond J. Safra Campus, Givat Ram, Jerusalem 9190401, Israel; ‡ Department of Medical Neurobiology (Physiology), The Hebrew University of Jerusalem, Hadassah Medical Faculty, Jerusalem, Israel 91120; § Department of Chemistry, School of Natural Sciences, Shiv Nadar Institution of Eminence, Greater Noida, Uttar Pradesh 201314, India

## Abstract

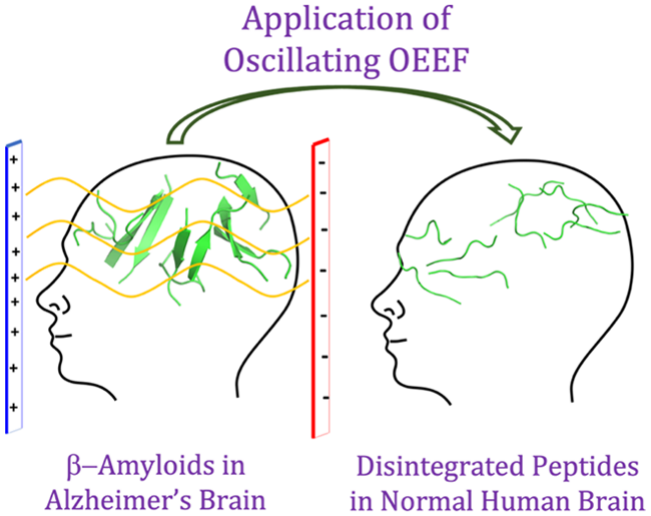

Alzheimer’s disease is one of the most common neurodegenerative conditions, which are ascribed to extracellular accumulation of β-amyloid peptides into plaques. This phenomenon seems to typify other related neurodegenerative diseases. The present study uses classical molecular-dynamics simulations to decipher the aggregation–disintegration behavior of β-amyloid peptide plaques in the presence of static and oscillating oriented external electric fields (OEEFs). A long-term disintegration of such plaques is highly desirable since this may improve the prospects of therapeutic treatments of Alzheimer’s disease and of other neurodegenerative diseases typified by senile plaques. Our study illustrates the spontaneous aggregation of the β-amyloid, its prevention and breakdown when OEEF is applied, and the fate of the broken aggregate when the OEEF is removed. Notably, we demonstrate that the usage of an oscillating OEEF on β-amyloid aggregates appears to lead to an irreversible disintegration. Insight is provided into the root causes of the various modes of aggregation, as well as into the different fates of OEEF-induced disintegration in oscillating vs static fields. Finally, our simulation results are compared to the well-established TTFields and the Deep Brain Stimulation (DBS) therapies, which are currently used options for treatments of Alzheimer’s disease and other related neurodegenerative diseases.

## 1 Introduction

The application of external and local electric fields has been proposed, in recent years, theoretically^1−8^ and demonstrated experimentally,^9−14^ as a means for controlling chemical reactivity for a variety of reactions, ranging from bond cleavage to cycloadditions, reactions of metalloenzymes, and hydrogen peroxide production.^1−12^ This includes changes in the equilibrium condition of chemical reactions^13^ and enhanced effectiveness of polypeptide proteolysis in the presence of the enzyme trypsin.^14^ In addition, oriented external electric fields (OEEFs) were shown to control product specificity, selectivity,^8,10a,10c^ and chiral discrimination.^1a,1b^

The present study goes beyond reactivity. It demonstrates that static and oscillating OEEFs control and regulate the order–disorder transformations of secondary structures of proteins/peptides aggregates. The order–disorder continuum in intrinsically disordered proteins (IDP) plays a role in various functions, such as cell-signaling, cell-cycle control, neurodegenerative disease, and many more functions.^15^ In particular, many neurodegenerative diseases are associated with misfolding of peptides/proteins, which perhaps lead to development of aggregates of misfolded proteins, acting as amyloid-like deposits.^16^ The exact mechanism of action and remedies of such diseases are still topics of intense research.

Alzheimer’s disease^17^ (AD), Parkinson’s disease^18^ (PD), Huntington’s disease^19^ (HD), Amyotrophic lateral sclerosis^20^ (ALS), transmissible spongiform encephalopathies^21^ (TSEs), etc. are some of the major neurodegenerative diseases, which are prevalent in modern society. Therefore, if IDP functions or misfolding of such peptides could be regulated by OEEFs, this may chart a new horizon for treating these debilitating conditions. As such, the present study attempts to deepen understanding of the regulation of insoluble peptide plaques by static and oscillating OEEFs.

To these ends we primarily focus here on a typical IDP, the Aβ-peptide, whereby the aggregation of protein fragments to β-sheets forms “senile plaques” which are likely to play a role in the initiation of Alzheimer’s Disease. We chose this specific Aβ-peptide due to the computational advantage of having a lesser number of residues than its counterparts which are formed by longer peptides or full-length proteins. At the same time, we aim to paint a general picture of OEEF mediated mechanistic exploration of misfolded peptides and their aggregation.

## 2 Brief Notes on AD and the β-Amyloid (Aβ) Peptide

AD is commonly observed at old age and constitutes a progressive neurodegenerative disease that causes memory loss, cognitive deterioration, and physical and behavioral dysfunction leading ultimately to death.^22^ Worldwide, AD contributes a total of 50–60% of nearly 50 million dementia patients.^23^ Despite this adverse impact of AD, a truly durable treatment is still unavailable. The current drug-based treatment can only delay the worst effects of dementia maximum up to one year.^24^ As such, a great number of research papers on AD are published every year, with the hope of gaining insight into potential preventive means for this severe neurological disease.

There exist several hypotheses concerning the various characteristics of the disease as well as its potential cures. The most prominent among these is the amyloid cascade hypothesis.^23,25,26^ According to this hypothesis, the extracellular aggregation of the β-amyloid (Aβ) peptide, known as a “senile plaque”, is the major cause of AD. Hence, in principle, the inhibition and clearance of this Aβ-peptide aggregation could be the most effective strategic therapy for the treatment of AD.^27^ Indeed, a recent study shows that the cognitive impairments of AD are correlated with the reduction in soluble Aβ_42_. As such, shifting the balance from plaque to disintegrated Aβ_42_ can be considered as a good therapeutic strategy.^28a^ Nevertheless, a recent communication^28b^ argues that several neurodegenerative diseases, e.g., the Parkinson disease,^28b^ are caused by the reduction of the amount of normal properly folded free protein rather than by the plaque formation per-se. Be this as it may, the plaque hypothesis remains prominent, and the decomposition of the plaque into properly folded normal proteins is still a major target. And if such a decomposition may lead to normally folded proteins, then a study of plaques decomposition is called for.

This Aβ-peptide usually originates from the transmembrane amyloid precursor protein, which appears predominantly in two different members, Aβ_40_ and Aβ_42_. Recent studies reveal that the Aβ_42_ variant forms oligomers, which are the major biological components for AD, and which serve as potential biomarkers in body fluids like serum and cerebrospinal fluid (CSF) for early diagnostics.^29^ Given this significance, researchers investigated and characterized the full-length Aβ_42_ peptide. However, the computational cost for an in-silico study of the complete peptide is very high.

**Scheme 1:**
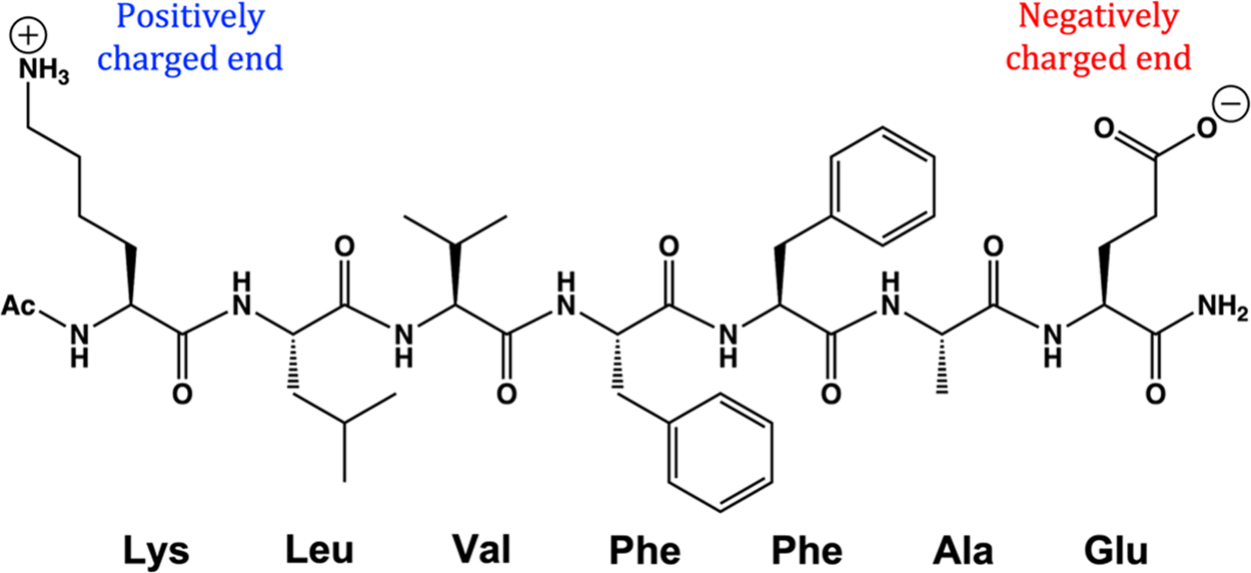
Chemical Structure of the Sequence *N*-Acetyl-KLVFFAE-NH_2_

In year 2000, Tycko et al. used solid-state NMR and subsequently reported the smallest ordered fibril forming segment of Aβ-peptide, which consists of seven residues (16 to 22) in the sequence *N*-acetyl-KLVFFAE-NH_2_.^30^ The chemical structure of this short peptide chain is shown in Scheme 1. These small peptides aggregate in an antiparallel β-sheet fashion to form highly ordered fibrils upon incubation in water. As such, this plaque constitutes an adequate computational model system in lieu of full-length Aβ-peptide. Hence, the present study focuses on the aggregation of this short peptide chain, and its disintegration under the application of static and oscillating OEEF.

A key step in AD amelioration has been associated with the molecular-level characterization of proper structures of disease-causing metastable protofibrils and mature fibrils. As such, researchers have gathered data obtained from numerous experimental techniques, such as NMR, ss-NMR, SEM, TEM AFM, FTIR, EPR, CD, etc., in an attempt to probe the molecular fold of these fibrils, their dimension, intermolecular packing including β-sheet formation, aggregation, and so on.^22d,31^

Alongside these experimental efforts, computational and molecular modeling studies have provided a wealth of mechanistic insight into the fibril’s structure and formation kinetics.^32^ Furthermore, the amyloids driven redox and the oxidative stress chemistry are also well studied.^22a,32d^ Thus, the details of such studies, which are covered in several outstanding papers and review articles,^32^ are not treated in the present study. The primary focus of this manuscript is the plaque, its mechanism of formations, and the best means for its irreversible decomposition.

## 3 Goals and Strategies

The TTFields therapy is a noninvasive medical approach, which harnesses the electric field to kill cancer cells in the treatment of solid tumors.^33^ Similarly, the Deep Brain Stimulation (DBS) is also a therapeutic technique, which has been employed for treatments of Parkinson’s disease, essential tremor, and dystonia.^34^ In contrast to TTFields therapy, DBS is an invasive technique wherein a pair of implanted electrodes deliver electrical impulses to specific targets of the brain, with an aim of ameliorating the clinical symptoms. These two fundamentally different therapeutic techniques form the basis of our motivation to use theory to investigate the impact and roots of noninvasive electric field effects on plaques that are related to brain disease.

The advances of computational resources enable us to investigate the characteristics of the β-amyloid peptide in the presence of static and oscillating OEEFs. We aim to provide a systematic study that may form a basis for using electric fields as potential therapeutic treatments of AD and related diseases. With this motivation, we conducted herein a comprehensive mechanistic study of the β-amyloid aggregation-disruption mechanisms under exposure to OEEFs. Thus, we employ long-time scale molecular-dynamic (MD) simulations of 1–1.5 μs duration in the presence of static and oscillating OEEFs at different strengths. We emphasize that the microsecond unit of time is the lifetime of molecular movements and not the actual time of the MD computations, which can last weeks.

These simulations aim at a systematic description of the following issues: (a) Gaining insight into the spontaneous formation of β-amyloid aggregation from the disordered polypeptide chains, using an OEEF-free simulation. (b) These amyloid clusters are then used to gauge the mode and propensity of aggregation at different OEEFs. As such, we apply both oscillating and static OEEF on well-organized/ordered antiparallel β-sheet clusters, which are shown to form spontaneously, and probe the disruption patterns which are brought about on the arrangement of these amyloid fibrils by the applied electric fields. (c) We then present a time-dependent correlation of our simulation results to the well-established DBS technique. (d) Finally, we demonstrate that our proposed oscillating-OEEF methodology is superior, and it can be corelated to TTFields therapy.

## 4 Methods

### System Preparation and MD Simulations

The initial structure of the β-amyloid polypeptide (cf. Scheme 1) chain was generated using the AMBER18 inbuilt Leap module.^35^ The protein force field ff14SB was used for treating the polypeptide chain,^36^ which was equilibrated to attain its relaxed conformation. This was followed by randomly placing 10 such relaxed polypeptide chains in a cubic TIP3P^37^ water box with dimensions 81.69 Å × 81.32 Å × 81.86 Å. Our simulation box contains 10,250 water molecules, and therefore, the concentration of peptides is 0.054 M [Molarity of peptides = (No. of solute particles or peptides/No. of water solvent molecules) × 55.5, where 55.5 is the molarity of pure water]. The overall charge neutrality of the system was ascertained.

After the completion of the system setup, the polypeptide–water mixtures were minimized in two steps; initially, solvent minimization was performed, and subsequently, the entire system was minimized without any restraint using 5000 steps of steepest descent followed by 5000 steps of conjugates gradient algorithm. The system was then gently heated from 0 to 300 K for 50 ps using the NVT ensemble, followed by use of 1 ns at the NPT ensemble at the target temperature of 300 K and a pressure of 1.0 atm using the Langevin thermostat^38^ and Berendsen barostat^39^ with a collision frequency of 2 ps and a pressure relaxation time of 1 ps. The so equilibrated systems underwent a further production run for 500 to 1200 ns, depending on the system requirements.

These runs used a multitrajectory approach in which the simulation was restarted at a random velocity after completion of each 50 ns duration. In this manner, the trajectory visits the various clustered minima without bias. All productive MD runs were divided into two groups: (i) in the presence of either static or oscillating OEEF and (ii) in the absence of OEEF. The magnitude of OEEF and the applied frequencies are reported in the Results and Discussion section.

The Monte Carlo barostat^40^ was used during all production MD simulations. Moreover, replica simulations were performed to ensure the consistency of the obtained results. The SHAKE^41^ algorithm was employed to constrain the hydrogen bonds, while particle mesh Ewald (PME)^42^ and appropriate cutoff distances (12 Å) were used to treat the long-range electrostatic and van der Waals forces, respectively. We systematically used Periodic Boundary Conditions (PBC) in all the simulations to remove the sharp edge problem of the simulation box.^35^ Furthermore, we scale the intensity of the applied external electric field using the AMBER in built keyword ‘efn = 1’. All MD simulations were carried out in the GPU version of the AMBER18 package.^35^ The CPPTRAJ module of the AMBER18 was used to analyze all the results. The PYMOL software package was mostly used to draw all figures. The energetics of the system were calculated using the MM/GBSA method.^43^

## 5 Results and Discussion

### 5.1 Aggregation Study of β-Amyloid Peptides in the Absence of OEEF

Each of the *N*-acetyl-KLVFFAE-NH_2_ chains (Scheme 1) has a large dipole moment (μ = ∼125 D), which originates in the charge separation in the two ends of the peptide chain (cf. Scheme 1). Having this high dipole moment, different chains will be mutually attracted by dipole–dipole interactions, as well as by chain–chain hydrogen bonds (H-bonds). This is indeed the scenario that emerges from the following MD simulations in the absence of an OEEF.

**Figure 1:**
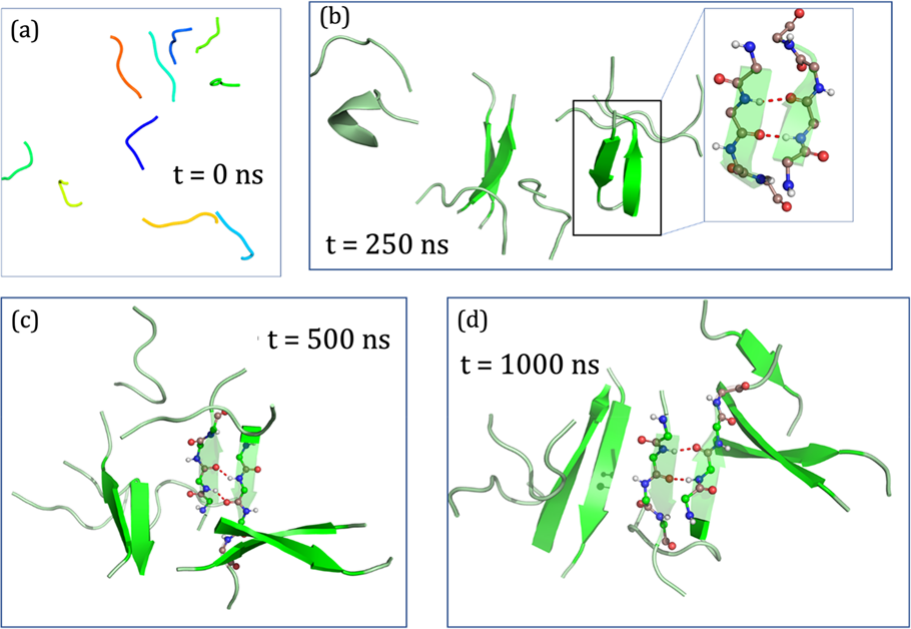
Snapshot of 10 randomly ordered β-amyloid peptides (described in Scheme 1) at different time scales of the MD simulations, resulting the eventual formation of β-amyloid aggregation. The time scale increases from (a) to (d). The arrows indicate the direction of each chain. The H-bonding between the chains during aggregation is depicted in dotted lines.

The mechanism of aggregation was elucidated using a microsecond-long MD simulation of 10 randomly ordered β-amyloid peptides (cf. Scheme 1). Indeed, aggregation of β-amyloid peptides, which started to a modest extent, increased gradually with the course of the simulation. The representative snapshots from the different time frames are shown in Figure 1.

As can be seen from Figure 1, initially, the system involved disordered polypeptides where the amyloid units were far apart from each other at *t* = 0 ns (Figure 1a) and possessing no secondary structures. However, as the simulation progresses, the chains gradually approached one another and transformed into an antiparallel β-sheet (see 250, 500, and 1000 ns), which involves primarily dipole–dipole interactions, augmented by H-bonding interactions in Figure 1c and 1d. During the longer simulation time (of ∼1000 ns) these β-sheets are perfectly arranged in a “cross” β-sheet fashion or “hairpin”-like structure which were reported earlier^31a,31b^ as the starting structures for the formation of “senile plaques”, which are stabilized by H-bonds and interchain dipole–dipole interactions (the Supporting Information (SI) includes a brief description of the time evolution of the peptides’ secondary structure, from the disrupted random coils (cf. S.1. and Figure S1)).

**Figure 2:**
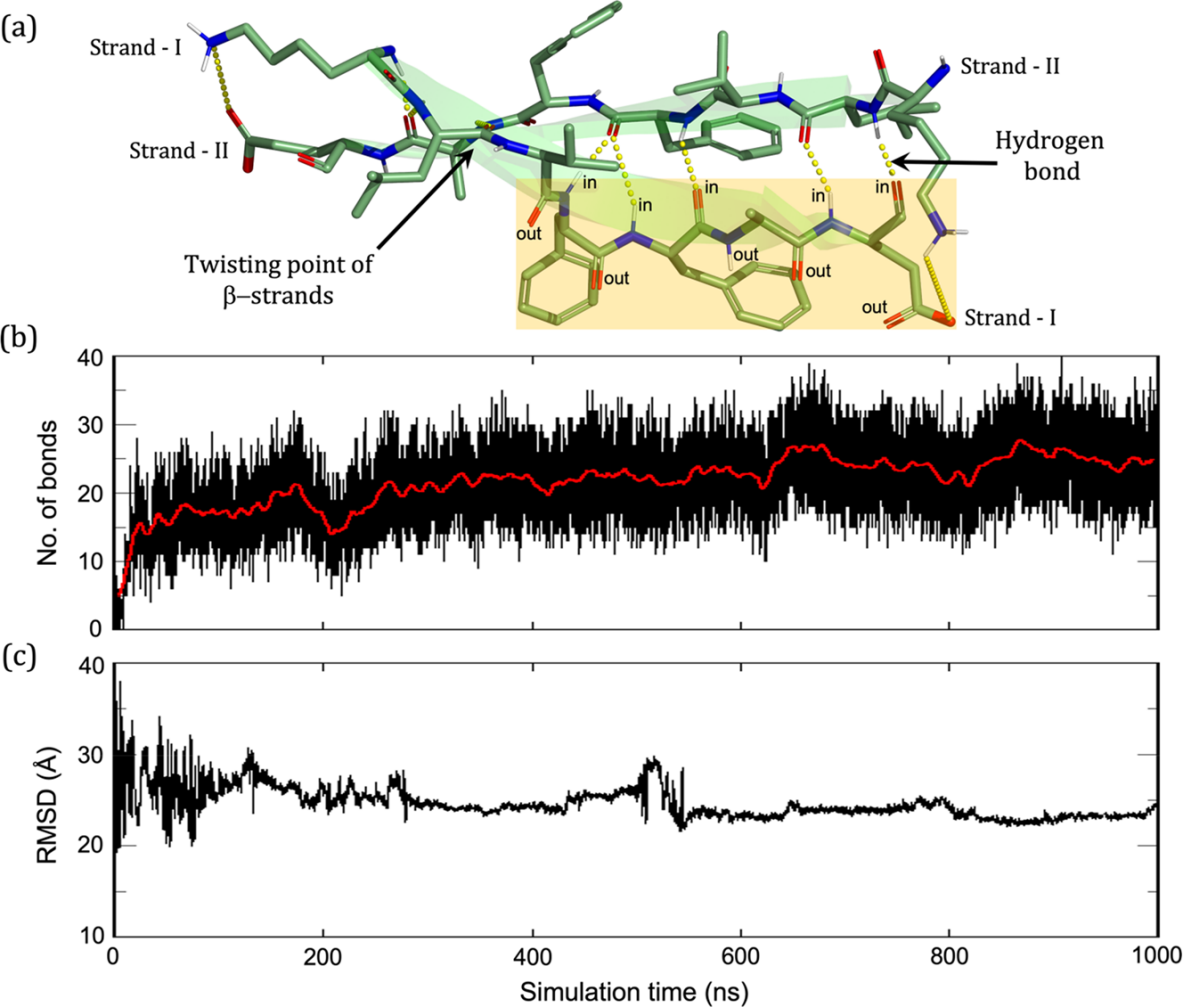
(a) Hydrogen bonding (“in” and “out” types) between strands, during the formation of the observed antiparallel β-sheet. The yellowish region describes the hydrogen bonding in “in” and “out” fashions; “in” H-bonds involve interchain interactions, while “out” ones are available to interact with another peptide chain and lead to a growth of the plaque. (b) Evolution of the number of hydrogen bond among the β-amyloid peptides with progress of the simulation. (c) RMSD plot of the backbone atoms for the entire trajectory and showing thereby the order created by reference to the starting frame.

Let us now discuss the role of hydrogen bonding and dipole–dipole interactions among the peptide chains in developing and attaining the stability of the resultant antiparallel β-amyloid aggregate. Figure 2a depicts the hydrogen bonds formed by two antiparallel β-strands arranged to form the β-sheet. We also highlight the atoms of a specific β-strand that participates in hydrogen bonding in an “in” and “out” fashion. Atoms in the “in” orientation are already engaged in a hydrogen bond with another β-strand, while atoms in the “out” orientation are available to interact with another incoming β-strand. Furthermore, the quantitative description shown in Figure 2b confirms that the number of hydrogen bonding increases as the simulation progresses, thus implying the formation of more β-sheet-like secondary structures. It follows therefore that the seminal β-sheet will interact successively with other peptides, and thereby outgrow the existing one.

Similarly, as stated above, a single peptide chain possesses a very large dipole moment (μ = ∼125 D). Therefore, the peptides will interact among themselves in an antiparallel fashion to achieve increased stability via dipole–dipole interactions, and as such will minimize the total dipole moment of the aggregate. Consequently, the global dipole moment of the 10-peptides aggregate is reduced to ∼166 D, which is significantly smaller than the sum of the dipole moments of all peptide chains in the aggregates (Figure S2 of S.2. in the Supporting Information depicts the electrostatic field lines generated by each peptide chain and shows further evidence of the strong dipole–dipole interaction in the antiparallel clustering).

In addition, Figure 2c provides further computational evidence for the stability of aggregation of the random peptide chains. Thus, examination of the RMSD plot reveals that, during the first 300 ns, the backbone atoms display high fluctuations around their mean positions, due to random movements of the chains away from one another. Subsequently, the fluctuations gradually subside, and the backbone atoms appear to be stabler and indicate that the system converged to a definitive antiparallel β-sheet aggregation.

As such, Figure 2 establishes that, in the absence of an external electric field, the β-amyloid peptides can efficiently and spontaneously assemble into antiparallel β-sheets, which is the onset for the development of the “senile plaques.”

### 5.2 Can OEEF Prevent the β-Amyloid Aggregation?

The role of large dipole–dipole interactions in the absence of an OEEF suggests that the amyloid aggregations should be highly sensitive to OEEF. We therefore focused on finding the optimal OEEF that can inhibit the β-amyloid aggregation. Accordingly, we applied external electric fields oriented along the *x*-axis starting from a very low strength of 0.01 V/Å keeping the same initial coordinates of the system as in section 5.1. We found that this OEEF slows down the rate of aggregation relative to the OEEF-free situation. Thus, while the used electric field is low, it nevertheless disrupts the spontaneous aggregation of antiparallel β-sheets. However, the propensity for aggregation, at this low field, was still maintained on the microsecond time scale. The representative snapshots at different time scales during the MD simulations at 0.01 V/Å are shown in Figure S5 along with the DSSP program secondary structure plot (similar to Figure S1a).

**Figure 3:**
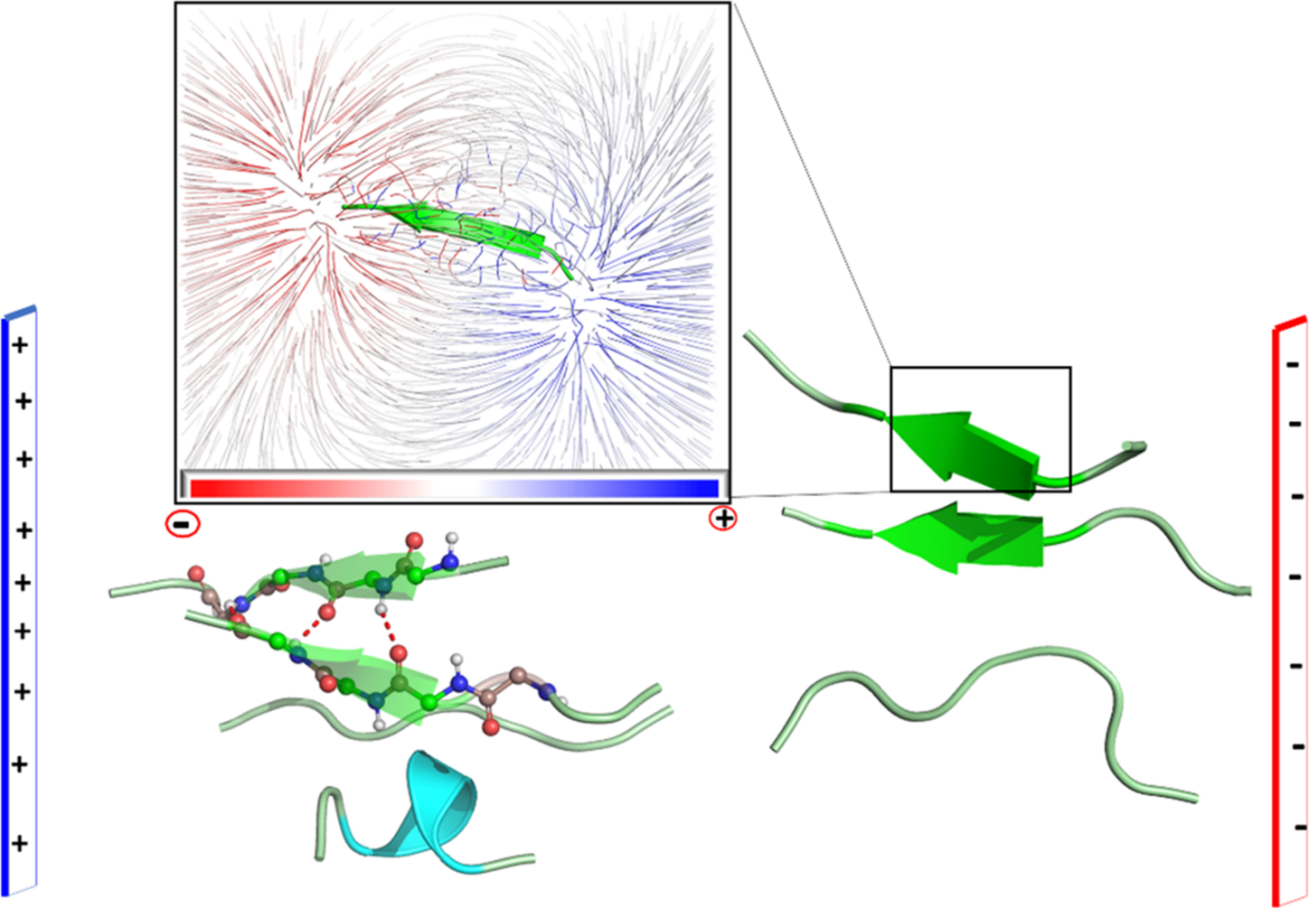
A snapshot which reveals the behavior of β-amyloid peptides in the presence of OEEF of strength 0.02 V/Å after 1000 ns of simulation time. The inset shows the electric field lines due to a monomer of amyloid fibril. The directions of two chains on the righthand side show a parallel arrangement.

Upon increase of the field strength to 0.02 V/Å, the inherent property of aggregation of β-amyloid peptide chain was significantly reduced. And furthermore, the peptides acquired a less defined secondary structure as shown in Figure 3 (cf. Figure S6, for detailed snapshots). In addition, as shown in Figure 3, in the presence of OEEF, the parallel β-sheet becomes the dominant arrangement and is preferred over the antiparallel β-sheet. This is emphasized by the directions of the arrows of two chains in the right-hand side of Figure 3 (see also Figure S7).

The key question at this point is how does the OEEF stabilize the growth of the parallel β-sheet instead of the antiparallel one? The interactions of peptides as well as their relative orientations depend on the strength of the applied OEEF (F_*x*_) and the respective dipole moments (μ_*x*_) of the two aggregate modes. This follows the expression in eq 1, where the field is used with units of V/Å and the dipole moment in Debye, and the sign of *ΔE* depends on the relative orientation of the field and the dipole moment:^10e,44^
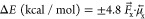
1

Furthermore, as demonstrated by Bretislav,^45^ the OEEF will also orient the peptide chain along the field’s axis. Using an arbitrarily direction *x*, eq 1 shows that a field F_*x*_ will orient the dipole moment of the peptide in an opposite direction along the field’s axis and will stabilize thereby the respective orientation of the peptide.

**Figure 4:**
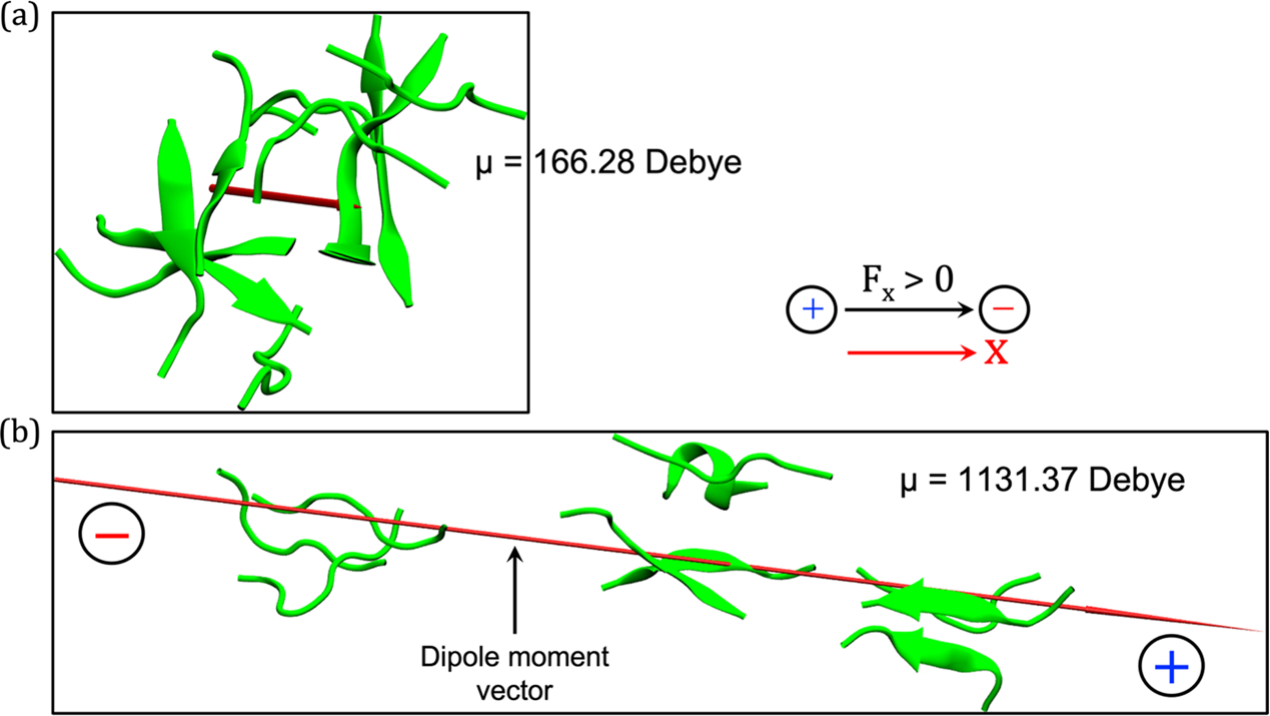
Representations of the dipole moment vectors: (a) In an antiparallel β-amyloid aggregation obtained in the absence of OEEF and (b) in a parallel β-sheet formed in the presence of OEEF of strength 0.02 V/Å. In both cases, the scale of the dipole vectors is proportional to their values. The black and red arrows, on the right-hand side, show the direction of OEEF and the Cartesian axis, respectively. For autocorrelation plots refer to Figure S9 (in the SI).

As shown in Figure 4, the dipole moment of an antiparallel arrangement in the absence of an OEEF (Figure 4a) is much smaller than the aggregated dipole moment of the parallel peptide chains (Figure 4b) in the presence of OEEF. Thus, the dipole moment of the parallel-chain arrangement is huge (1131.4 D), by comparison to the dipole moment of the antiparallel arrangement in the no-field simulations. As such, the parallel β-sheet is stabilized by the OEEF (by ca. 108 kcal/mol), despite the unfavorable peptide–peptide dipole interactions. At the same time the antiparallel arrangement is stabilized mostly by dipole–dipole interactions and interchain hydrogen bonds, while its interaction with the OEEF is approximately vanishing (since one chain interacts favorably with the field and the other unfavorably). As such, the antiparallel arrangement is less stable in the presence of the OEEF compared with the parallel arrangement.

In addition, the nonaggregating nature of peptide chains (in either orientation) in the presence of OEEF can be substantiated by the nature of the RMSD plot (cf. Figure S8). A careful analysis of the plot indicates that it closely resembles the RMSD of the first 100 ns of the zero-field simulation, where no aggregation was observed (cf. Figure 2c). This observation shows that, in the presence of an OEEF of 0.02 V/Å, the chances of β-amyloid aggregation are dismal.

In a nutshell, we can conclude that the proximity between the peptide chains is markedly reduced in the presence of OEEF, thus showing a destruction of the β-amyloid aggregation. Consequently, the antiparallel aggregate is replaced by a loose pack of parallel peptides that are held together by the OEEF due to their overall huge dipole moment (cf. Figure 4), which is at least a sum of the individual chain’s dipole moments (should a polarizable force-field were to be used).

### 5.3 Application of Static OEEF to the β-Amyloid Aggregation

**Figure 5:**
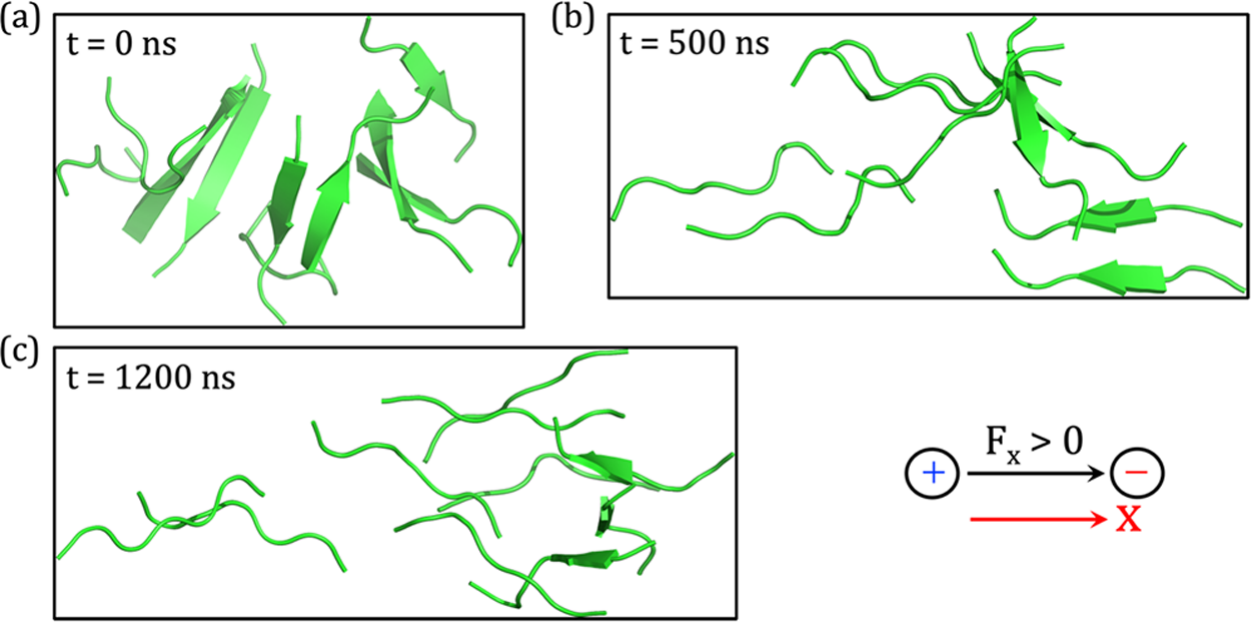
Snapshots of β-amyloid aggregates at different time scales in the presence of a static OEEF of strength 0.02 V/Å. The time scale increases from (a) to (c). The “black arrow” denotes the direction of the positively oriented OEEF vector along the *x*-axis (red arrow) direction.

The preceding section demonstrates that the presence of OEEF prevents the β-amyloid aggregation. However, there is still an open question: Is the same OEEF strength (0.02 V/Å) sufficiently capable of disrupting a well-grown aggregated form of β-amyloid peptides? To answer this question, we applied an OEEF of strength 0.02 V/Å on the aggregated form of peptides and performed the MD simulations. The initial coordinates for this simulation are taken from the end result of the no-field simulation, which is shown in Figure 5a.

A thorough analysis of the MD trajectory shows that the applied OEEF significantly destabilizes the β-amyloid aggregation. As seen in Figure 5b, the chains start separating from each other at ∼500 ns of the simulation. We further extended our simulation to 1200 ns to ensure complete disintegration and discovered that all β-strands change to their natural primary peptide chains with no sign of aggregation (cf. Figure 5c). Hence, we may conclude that a moderate OEEF of 0.02 V/Å suffices to destroy the spontaneously formed and well organized antiparallel β-amyloid aggregation to random coils in their native states.

However, if we perform a time-dependent correlation based on our previous simulation, we can see that the time required to form the spontaneous antiparallel β-amyloid aggregation without a field is almost the same (on a microsecond scale) as the time required to break the aggregate under the OEEF of strength 0.02 V/Å. Thus, one may have been tempted to argue that the OEEF must be applied for long periods of time in order to maintain the disrupted amyloid aggregation. This, however, is not a good idea for treatment of humans. Hence, at this point, we have two options: either increasing the field strength, so that the aggregation breaks early in the simulation scale, or applying an oscillating OEEF with the same strength. Since increasing the OEEF intensity may be harmful, we chose the second choice as discussed in the following sections.

### 5.4 Application of Oscillating OEEF to the β-Amyloid Aggregation

The prime goal of simulating the oscillating OEEF-effects is to find an optimum frequency which is suitable for an application within the human brain. Thus, we begin our investigation with a very low microwave frequency in the GHz band. We chose this band because all living animals in today’s world, including humans, are constantly exposed to microwave frequency which is widely used in telecommunication, radar, navigation systems, and other applications. Accordingly, we attempted to gauge the range of frequencies that works for the β-amyloid disaggregation in the presence of OEEF = 0.02 V/Å.

**Figure 6:**
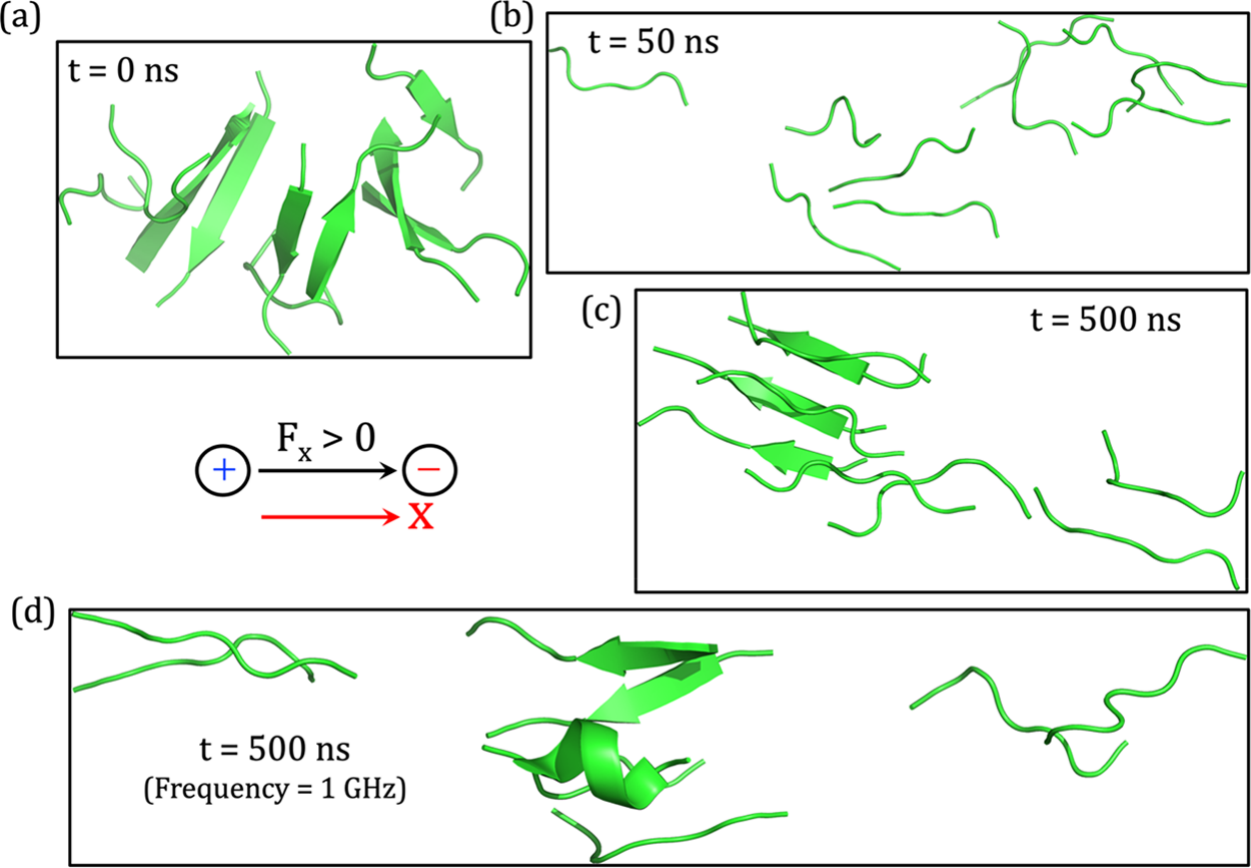
Snapshots of β-amyloid aggregates at different time scales in the presence of an oscillating OEEF of strength 0.02 V/Å with frequencies of 0.1 GHz (parts a–c) and 1 GHz (part d). The duration increases from (a) to (c). Similarly, (d) is evolved from (a) under 1 GHz frequency. The “black arrow” in between the panels denotes the direction of the positively oriented OEEF vector along the *x*-axis (red arrow).

We started these simulations using a frequency of 0.1 GHz in Figure 6. We found that, by comparison to the static field, in the presence of an oscillating electric field the disruption of β-amyloid occurs very fast. All results are summarized in Figure 6a to 6c.

### 5.5 Irreversibility of the Oscillating OEEF Effect

It is gratifying to note that the oscillating OEEF (0.1 GHz) requires only 50 ns of simulation time to completely destroy the existing pairs of antiparallel β-sheet and convert them to random coils. Surprisingly, after 500 ns of simulation time, these random peptides remain widely separated from one another, with the exception of a pair that still exhibits slightly parallel β-sheet characteristics. Additionally, this finding is very similar to the end result obtained in section 5.2, where we saw how static OEEF prevents the spontaneous development of β-amyloid aggregation from random peptide chains. However, an oscillating OEEF requires a much smaller time scale (Figure 6a) to destroy the plaque compared with the time scale required by use of a static OEEF (50 vs 500 ns).

To explore the effect of the oscillating frequency of the OEEF, we increased the frequency in Figure 6d to 1 GHz at the same OEEF strength of 0.02 V/Å. Similar to the previous simulation, in Figure 6a–c, Figure 6d reveals that the amyloid aggregation gets broken down very quickly. Moreover, at the end of the 500 ns simulation time in Figure 6d, the propensity of β-sheet formation becomes negligible.

A visual comparison of the snapshots obtained after 500 ns of simulation time at both frequencies shows that a moderate increase in frequency up to 1 GHz may result in greater separation of the random coils. Thus, we can expect that further increase in frequency may produce better outcomes. At the same time, these two frequencies are already satisfactory and sufficient proof for the destruction of β-amyloid aggregation. Note that, in all cases of oscillating OEEF simulations, we conducted the simulation up to 500 ns because the RMSD converges within a few nanoseconds, without any observable conformational transition following the β-amyloid breakdown at ∼50 ns (cf. Figure S10).

Similarly, it is important to reiterate that whereas the breakage of the plaque, under an oscillating electric field, requires only 50 ns, the static OEEF (cf. section 5.3) requires an increased time to 500 ns. Hence, we may conclude that oscillating OEEFs of ∼0.02 V/Å of small microwave frequencies, ranging from 0.1 to 1 GHz, can efficiently separate the well grown β-amyloid aggregation to the random collection of peptides.

Let us try to comprehend why and how this superior disruption rate, of the β-amyloid, is achieved by the oscillating OEEF. We have already mentioned in the foregoing sections that a single peptide chain has a significant dipole moment (μ) along its chain (say the *x* axis). As such, when the static OEEF is applied to a bunch of peptides or their cluster, the peptide chains become oriented along the direction of the applied OEEF and achieve stability with a stabilization energy, *ΔE* [*ΔE* (kcal/mol) = 4.8 μ_*x*_·F_*x*_]. By contrast, in the application of oscillating OEEFs, whereas the first phase of the oscillating field (say, positive phase) stabilizes the parallel peptides arrangement, as does the static field, this stability becomes completely disrupted as soon as the oscillating field changes phase (say, the negative phase). Thus, during the phase change, the cluster of parallel chains will experience augmented destabilization: (i) repulsion between the dipoles of the interacting parallel chains, as well as (ii) repulsion of the entire aggregate by the electric field. Hence, breakdown of β-amyloid aggregation will occur rapidly whereas rearrangement to the antiparallel plaque will take longer, compared with the effect of a static OEEF.

#### 5.5.1 Fate of Disrupted β-Amyloid Peptides Upon Removal of the Oscillating OEEF

**Figure 7:**
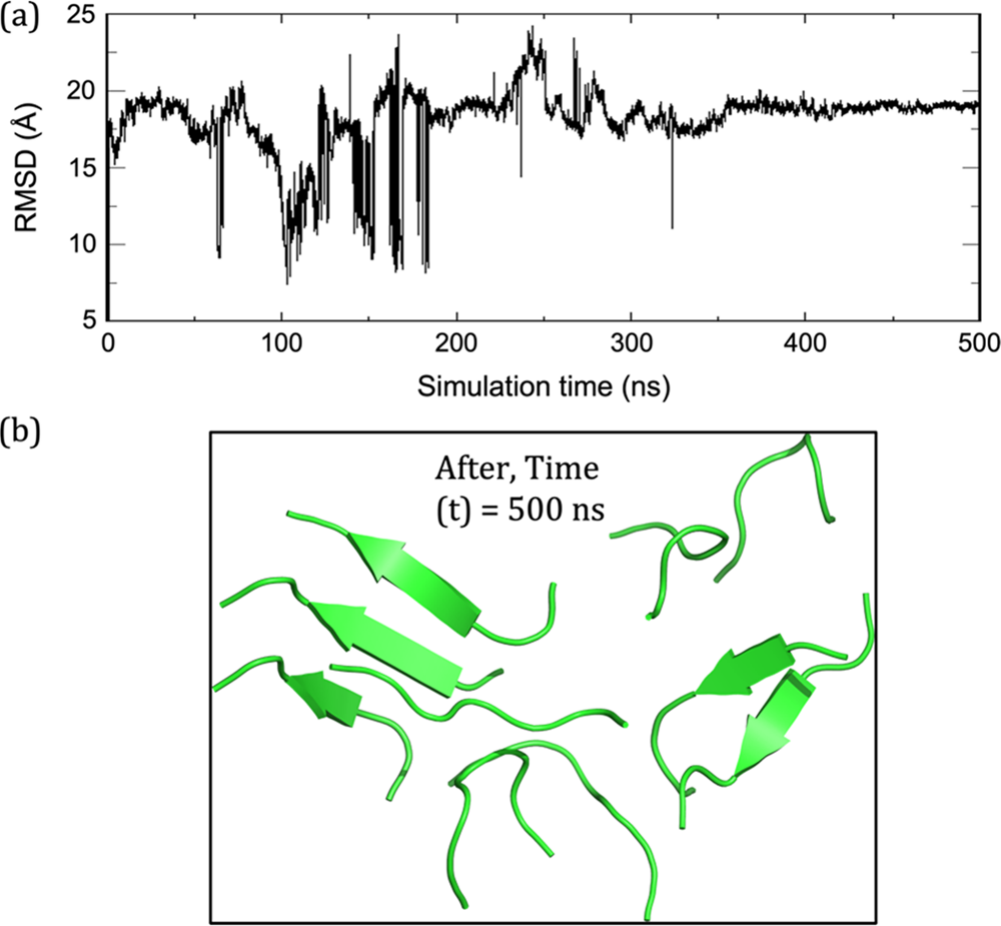
Behavior of the parallel arrangement after removal of the oscillating OEEF: (a) RMSD plot for the backbone atoms with reference to starting frame for the entire trajectory. (b) Snapshot showing the behavior of peptide chains at 500 ns upon removal of oscillating OEEF of strength 0.02 V/Å and frequency 0.1 GHz. Note that the plaque disintegrated and will remain so beyond 500 ns. See also Figure S11 for the RMSD of the 1.5 μs MD.

The efficient decomposition of the plaque by the oscillating OEEF raises a key question: Do the well-separated peptide chains return to the original conformation of β-amyloid aggregation after turning off the oscillating OEEF? To answer this question, we used the system obtained after 500 ns of simulation in the presence of oscillating OEEF of strength 0.02 V/Å with a frequency of 0.1 GHz and allowed it to relax by turning off the oscillating electric field. The simulation converged very quickly (below 300 ns) without any change in the disintegration of the plaque. This is shown in Figure 7, which presents the RMSD convergence in (a) and the still disintegrated plaque in (b) at 500 ns. In order to test the consistency of the obtained result, we extended the simulation up to 1.5 μs and observed a similar conformation of peptides that was seen at 500 ns (cf. Figure S11 for the corresponding RMSD plot).

Importantly, as can be seen vividly in Figure 7b, random coil polypeptides do not spontaneously return to their antiparallel cross β-sheet amyloid aggregation upon removal of the OEEF. Instead, they just prefer to stay in the random conformation with a little grown parallel β-sheet. As such, it can be concluded that the random coil or half grown parallel β-sheet that was formed under oscillating OEEF do not spontaneously return to form the ‘β-amyloid aggregation’ upon removal of the oscillating electric field.

This important conclusion raises the question of whether this irreversibility may be augmented by chemical changes or the dissociation of an amino acid’s side chain. To further respond to this fundamental issue, we thoroughly studied the structural and chemical changes of a little-grown parallel β-sheet, which was developed after 500 ns of an oscillating OEEF simulation at 0.1 GHz (OEEF = 0.02 V/Å) and was sustained after turning the field off (cf. Figure S3). This comparative observation demonstrates that the irreversibility is augmented by chemically related mechanisms, specifically in the disrupted interpeptide hydrogen bonding. Furthermore, some contribution may be expected from steric clashes of the amino acid’s side chain-side chain interactions during complete reversal of the chain. A brief discussion is given in the SI (cf. S.3.). Chemical reactions, e.g., proton transfers,^3,14^ are likely to occur as well and disrupt thereby the reversibility of plaque reformation. However, these latter effects cannot be probed by classical MD simulations. The role of hydrogen bonding has also recently been used to describe the piezoelectric responses of oligomeric peptides.^46^

### 5.6 Prospects of Irreversibility with Oscillating Fields

So far, we have seen that the changes in the amyloid aggregation caused by oscillating OEEF are irreversible. However, it remains unclear whether this observation is consistent or merely an accidental result. We therefore carried out a set of simulations using four different frequencies with 10-fold increments.

**Figure 8:**
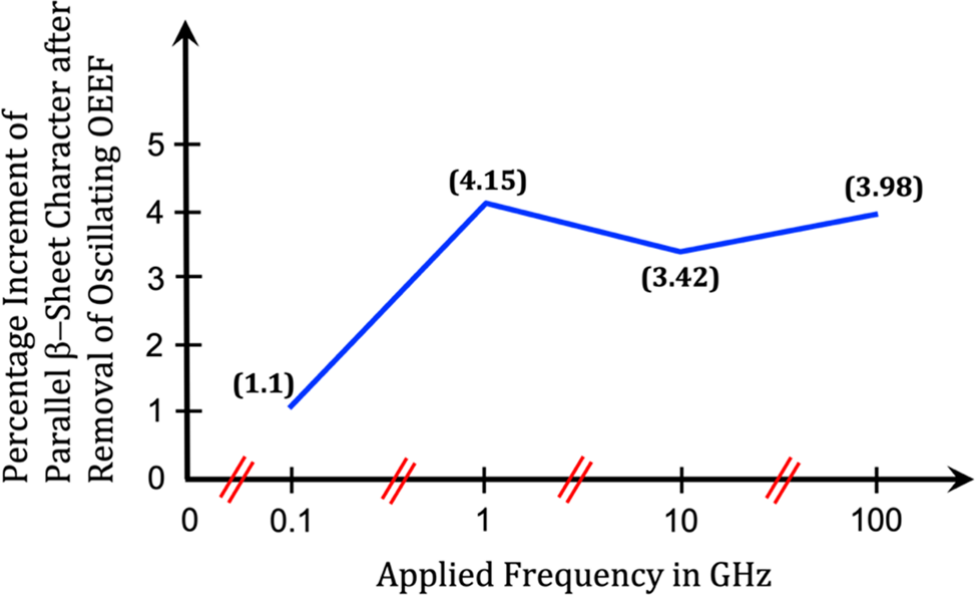
A plot showing the percentage increment of parallel β-sheet character upon removal of the oscillating OEEF. Values in parentheses near each point in the plot shows the exact residual percentages. The scale in *x*-axis is broken after every point.

Thus, each of the four simulations was performed with an OEEF strength 0.02 of V/Å at frequencies of 0.1, 1.0, 10.0, and 100.0 GHz: hence exhibiting a range of 3 orders of magnitude in the frequency. In all cases, we first ran the simulations for 500 ns at the respective frequencies, in the presence of the OEEF, and subsequently allowed them to relax for another 500 ns by removing the oscillating OEEF. Figure 8 depicts a quantitative plot derived by analyzing these trajectories. It is seen from Figure 8 that after the oscillating OEEF was removed, the percentage of average parallel β-sheet character increased very slightly in all cases and reached ca. 4% (cf. Figure 8), without signs of antiparallel sheets formation. As such, this finding paints a picture of the likely steady irreversible nature of broken β-amyloid aggregation obtained with oscillating OEEFs.

Our treatment addressed the fundamental questions and laid the physical basis for the spontaneous occurrence of antiparallel β-sheets, and the formation of parallel β-sheets only under OEEF. Nevertheless, the Tycko group^31c^ found evidence that the other form of amyloid peptide, *Aβ*_40_, can organize in a parallel arrangement (and presumably induce AD). Such findings appear also in recent studies,^47^ which suggest a heterogeneous scenario of both forms in, e.g., the solid state (X-ray structures),^47b^ as well as an antiparallel arrangement as the starting point^47a^ for many or majority of the amyloid aggregation processes. It follows therefore that the apparent irreversibility of amyloid aggregation found here, after cessation of the oscillating OEEF, for peptides in aqueous solution (wherein the zwitterionic form is stable), appears to be highly significant. This can be contrasted with our findings (cf. Figure S12) that the neutral species of the same randomly ordered peptides do not self-aggregate upon long time scale simulation.

Furthermore, a careful evaluation of the plot in Figure 8 reveals that the growth of the parallel β-sheet following the removal of the OEEF is very slow, with only 1.1% of increment after 500 ns of simulation time for the frequency of 0.1 GHz. In addition, parallel β-sheets are energetically higher than the antiparallel ones (cf. S.4. and Figure S4, for detailed energetics) and the formation of such β-sheets in the absence of OEEF is found in our study to be nonspontaneous. As a result, it is very unlikely that the reaggregation would occur fast, and if at all, it will require a significantly longer time to morph into a fully fledged β-amyloid aggregation.

In summary, our results demonstrate that the use of an oscillating OEEF to destabilize β-amyloid aggregation is an irreversible process that does not revert easily to its aggregated form when the OEEF is removed.

### 5.7 Correlation of OEEF Procedure to TTFields Therapy and DBS Technique

In addition to the description at section 3, the TTFields therapy uses a frequency range of 100 to 500 kHz of the applied electric field to selectively kill cancer cells without destroying the normal ones.^33^ Similarly, our MD results demonstrate that an OEEF of frequency 0.1 to 1 GHz achieves noninvasive destruction of the aggregated misfolded Aβ peptides.

In the case of the DBS technique, when the electrical impulses of the implanted pair of electrodes are turned on, usually trains of 60 μS pulse-width, at a frequency of 130 Hz, they pass through the targeted area, and the patient gradually begins to experience relief of his/her motor symptoms.^34^ However, the symptoms will recur, and a single application of an electrical impulse is insufficient for a long-lasting relief. As a result, the patient must use it on a regular basis to improve symptoms and health.

Let us now correlate the above DBS procedure with our obtained molecular results. When we conduct a time-dependent correlation of our simulation data, we can see that the spontaneous generation of β-amyloid aggregation requires about 1 μs of simulation time. On the other hand, the so-formed amyloid becomes broken in only a 50 ns simulation time in the presence of oscillating OEEFs. Thus, the breakage of the amyloid aggregation is nearly 20 times faster than its formation time span. Furthermore, if we remove the OEEF, the disrupted β-amyloid aggregation does not revert back (at least for 1.5 μs long MD simulation) to its initial state, which further demonstrates that the disintegration process is irreversible.

Finally, according to the description in section 5.6, the probability for the generation of parallel β-amyloid aggregation is minimal, and it will take many times longer than the duration of its normal formation, as discussed in section 5.1. Taking all these points into consideration, we may conclude that a periodic application of oscillating OEEF may serve as a promising therapeutic treatment for Alzheimer’s disease and related neurodegenerative disease.

## 6 Conclusion

The goal of this manuscript was to explore the prospects of a potential therapeutic treatment for Alzheimer’s and similar neurodegenerative diseases that are caused due to amyloid-like deposits of aggregated misfolded proteins. The proposed therapeutic means involves an oscillating oriented external electric field (OEEF). Our findings show that the application of an oscillating OEEF on β-amyloid peptides can cause a few major changes: (i) prevention of the β-amyloid aggregate formation by noninvasive means; (ii) irreversibly driving the aggregated form to its native state of random coils; and (iii) these effects occur in the microwave band, which humans are exposed to on a daily basis.

By contrast, using a static OEEF requires a much longer time to break a well-grown β-amyloid aggregation, and the process is reversible with the same duration as the deaggregation. Therefore, the use of noninvasive oscillating OEEFs is deemed superior over the use of static OEEFs. A similar investigation of Parkinson’s disease, which is associated with the folding/unfolding of the α-synuclein protein, may reveal if the above conclusion is general.

Finally, we linked our findings to the established TTFields therapy as well as DBS technique, and we conclude that a periodic application of a well-optimized oscillating OEEF technique will open the door for a possible therapeutic treatment of Alzheimer’s and other neurodegenerative diseases. Clearly, whether the plaque is the root cause of the disease or not remains an interesting question. It might still be that the destruction of plaques simply increases the amount of “healthy” proteins which are properly folded, and brings about some relief from these neurodegenerative diseases. This leaves some fundamental questions for the future.

## Supporting Information Available

Comprehensive information on secondary structure evolution (DSSP plots), secondary structure distribution (Boltzmann distribution plots), root-mean-square deviation (RMSD plots), electrostatic behavior of the peptide chain, energetics of different conformations, and several detailed snapshots. (PDF)
