# Supplementary material for: How Can Static and Oscillating Electric Fields Serve in Decomposing Alzheimer’s and Other Senile Plaques?
Source: J Am Chem Soc. 2023 Feb 3;145(6):3543–53. doi: 10.1021/jacs.2c12305 (PMC9936589; doi:10.1021/jacs.2c12305)
Supplement: Supplementary file 1 — ja2c12305_si_001.pdf [file ja2c12305_si_001.pdf]

Supporting Information

How Can Static and Oscillating Electric Fields Serve in Decomposing Alzheimer's and Other Senile Plaques?

Surajit Kalita[1], Hagai Bergman[2], Kshatresh Dutta Dubey[3*] and Sason Shaik[1*]

1. Institute of Chemistry, The Hebrew University of Jerusalem, Edmond J. Safra Campus, Givat Ram, Jerusalem 9190401, Israel.
2. Department of Medical Neurobiology (Physiology), The Hebrew University – Hadassah Medical Faculty, Jerusalem, Israel 91120.
3. Department of Chemistry, School of Natural Sciences, Shiv Nadar Institution of Eminence, Greater Noida, Uttar Pradesh 201314, India.

Table of Contents:

S.1. Description of secondary structure evolution of peptides with time S2-S3

S.2. Description of electrostatic behaviour of peptide chains S3-S4

S.3. Irreversibility of peptides configurations after removal of oscillating OEEF S4-S5

S.4. Energies of parallel and antiparallel β–sheets S6-S7

Figure S1: DSSP and Boltzmann distribution plot S2

Figure S2: Electrostatic field lines of peptides S4

Figure S3: Comparative parallel β–sheets S4

Figure S4: Linear interaction energy (LIE) plots S6

Figure S5: Several snapshots with time evolution and corresponding DSSP plot S7

Figure S6: Several snapshots with time evolution S8

Figure S7: DSSP and Boltzmann distribution plot S8

Figure S8: RMSD plot for the static OEEF simulation S9

Figure S9: Autocorrelation Plot S9

Figure S10: RMSD plots for the oscillating OEEF simulations S10

Figure S11: Extended RMSD plot corresponds to Figure 7 S10

S.1. Description of Secondary Structure Evolution of Peptides with Time

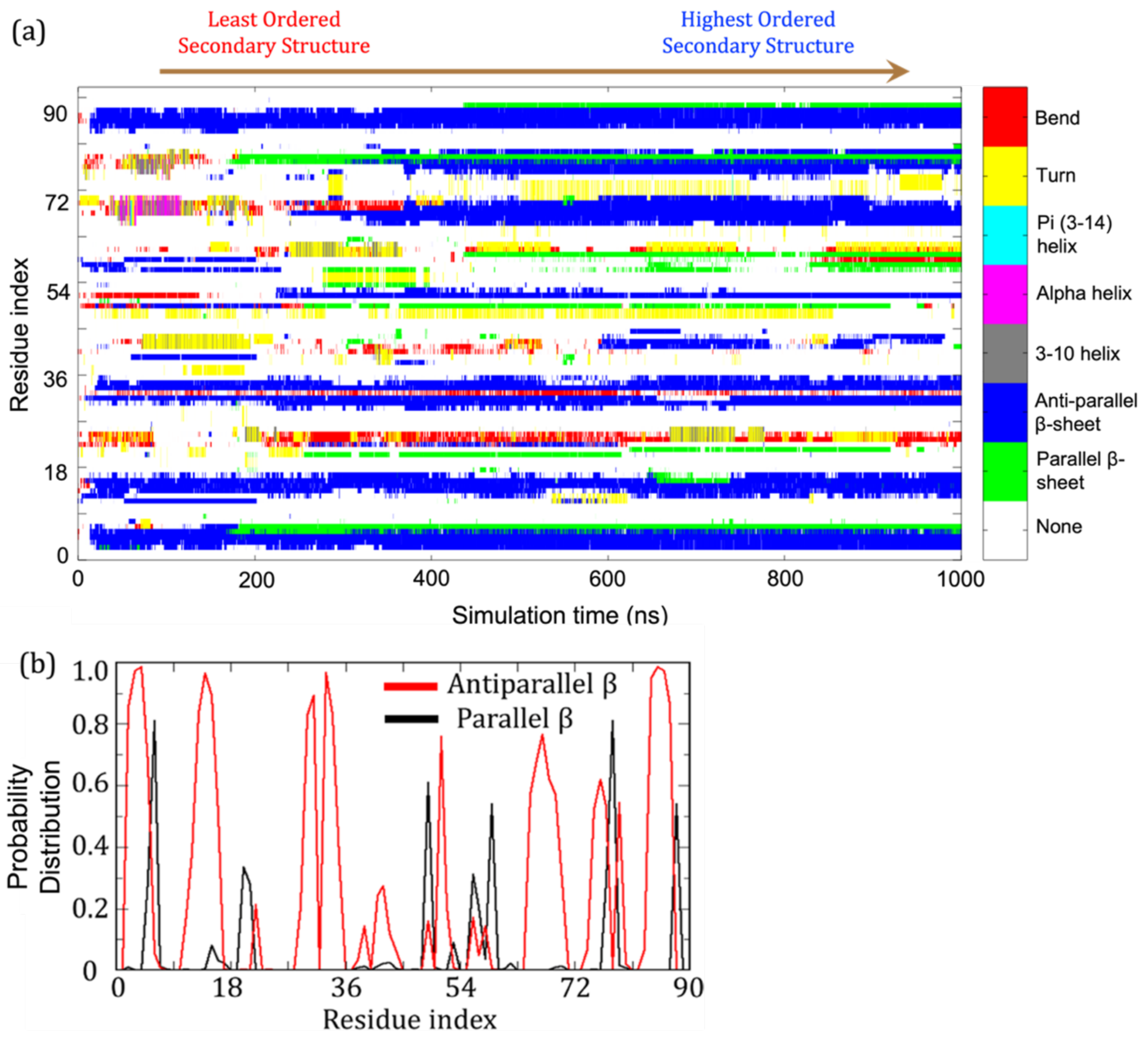

Figure S1: (a) Evolution of a secondary structure of 10 peptides with simulation time. Every 9-residue index in the y-axis signifies a single polypeptide chain. (b) Residue-wise Boltzmann probability distribution of parallel and antiparallel β-sheet. Every 9-residue index in the x-axis signifies a single polypeptide chain. Note that the initial 4 ns of equilibrium simulation data prior to recording the production MD is not included.

A clearer understanding of the evolution of a secondary-structure which replaces the disrupted random coils, can be achieved with the AMBER inbuilt DSSP program. In Figure S1a, we have attempted to understand the formation of various secondary structures using

different color codes. The width of each color band along the y-axis represents the natural propensity of that specific secondary structure. If no color code (white) is assigned to a particular residue, it means that that residue does not have any secondary structure at the inspected moment of time. Thus, as can be seen in Figure S1a, there is no color code for the polypeptide chain during the first few nanoseconds, *which indicates that they do not have any definite secondary structure at the start of the simulation*.

It is seen that these random coils are very prone to transformations to various secondary structures. A closer look at Figure S1a reveals that the 'blue' colour is the most prominent one throughout the simulation, and *it is represents the antiparallel β-sheet*.

In addition, we can also observe 'green' and 'yellow' as the second most visible colors in Figure S1a, which represent 'turn' and 'parallel *β*–sheet' respectively. We did not emphasize 'turn' secondary structure because we are interested in studying the formation of *β*–sheets.

In Figure S1b, we used the Boltzmann probability distribution plot to quantify the visual observation of parallel and antiparallel β–sheets corresponding to each peptide residue. Thus, it is clear that the antiparallel *β*–sheet character (in red) is the dominant secondary structure possessed by the majority of the peptide residues. Hence, our graphical analysis of the MD trajectory also corroborates to the pictorial result that shown in Figure 1 of main text.

S.2. Description of Electrostatic Behavior of Peptide Chains

As shown in Scheme 1 (in main text), there is a distinct charge separation in the peptide chain, with two oppositely charged residues at the extreme end. Thus, each peptide will behave as a single molecular dipole and will strongly couple with the applied OEEF. Figure S2 plots the electric field lines for the *β*–sheet, and shows that each strand of β–amyloid peptide possess a high dipole moment (μ = ~125 Debye), with the head of the strand being negative and the tail being positive. Clearly therefore, when these *β*-strands are put together in a cluster, they

mutually reorient in a head-to-tail antiparallel manner so as to lower the total energy of the aggregate.

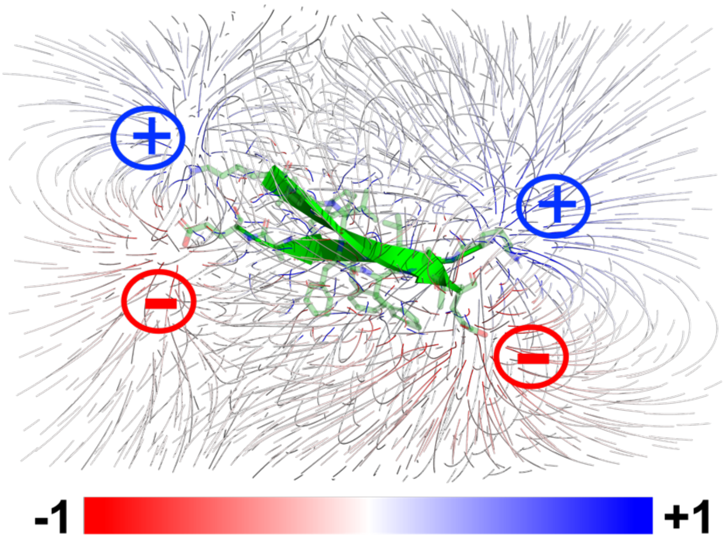

Figure S2: β−strands tend to orient in mutually antiparallel arrangements (*β*−sheet formation) to lower the electrostatic energy. The red-blue strip shows the characteristics of electric field lines generated by PYMOL incorporated APBS electrostatics plugin. Note that the initial dipole moment of a chain is 125 Debye, while the resultant dipole moment of both *β*−strands after forming antiparallel β−sheet is reduced to ~46 Debye.

S.3. Irreversibility of Peptides Configurations after Removal of Oscillating OEEF

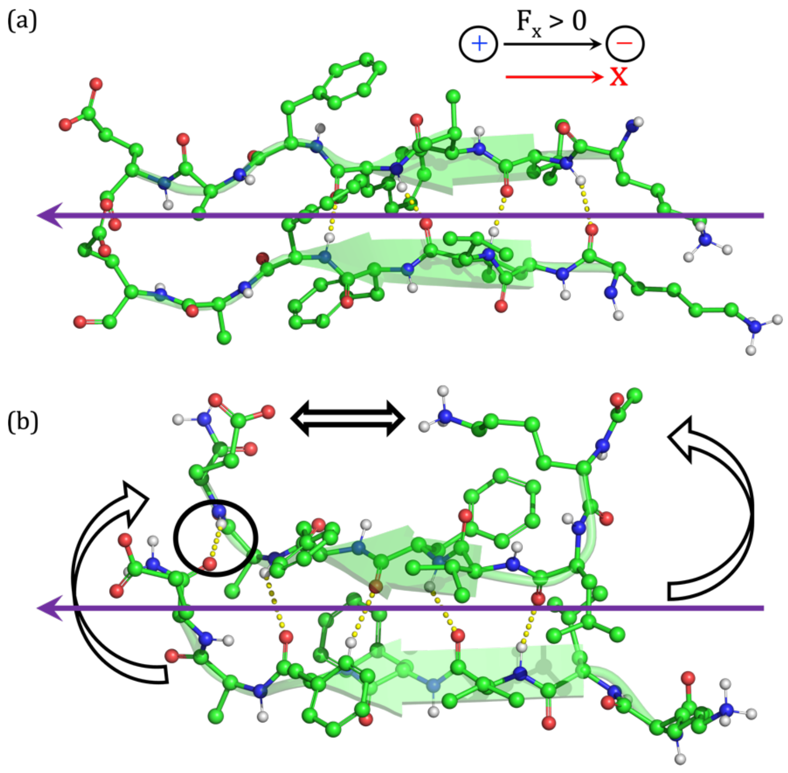

Figure S3: A little grown parallel *β*–sheet: (a) after 500 ns of simulation duration under an oscillating OEEF with frequency 0.1 GHz and strength 0.02 V/Å. (b) The same β–sheet after 500 ns of simulation time following field removal. The line describes an imaginary β–sheet axis passing between the chains. The curly arrows show the deviation of peptide chain from their original position. The double headed arrow denotes the probable sidechain-sidechain steric clashes. Yellow dotted lines are interpeptide hydrogen bonding. The 'black arrow' on the top of Figure a denotes the direction of the positively oriented OEEF vector (red arrow) along the x-axis.

It is essential to comprehend how irreversibility emerges in peptides configuration that is forms under oscillating OEEF. Thus, we identify a little-grown parallel *β*–sheet that is generated during simulation under oscillating OEEF. Thereafter, we have thoroughly studied the structural and chemical changes of the chains that occur upon removal of the oscillating OEEF. Figure S3 depicts the chemical and biological structure of the *β*–sheet for both cases.

Let us now construct an imaginary *β*–sheet axis (purple line, cf. Figure S3), which passes between both chains. In the case of Figure S3a, the applied OEEF and both chains are parallel to the β–sheet axis. On the other hand, Figure S3b demonstrates that in the absence of OEEF, both extreme ends of the chains are not parallel to the *β*–sheet axis. Instead, the chains are trying to deviate from their initial conformation by moving away from one another (see direction of curly arrows). Thus, it becomes clear that both chains try to separate from each other when the oscillating OEEF is removed, but interpeptide hydrogen bonding (see dotted yellow bonds) still holds the chains together. Furthermore, the hydrogen bond (black encircled) at the left end of Figure S3b shows how tightly the chains are bound, as the upper chain drags the bottom one along with it. As a result, both chains curl and deviate from their original positions. Hence, interpeptide hydrogen bonding plays the crucial role in irreversible nature of peptides configuration upon removal of oscillating OEEF.

Additionally, we observe the amino acid's sidechain-sidechain steric clashes (see double headed arrow in Figure S3b), which will be significant upon complete reversal of the peptide chain.

S.4. Energies of Parallel and Antiparallel *β*–Sheets

We investigated the energetic differences between the antiparallel and parallel β-sheets using various techniques such as MMGBSA calculation, Linear Interaction Energy (LIE) plot, and quantum-mechanical single point energy evaluation.

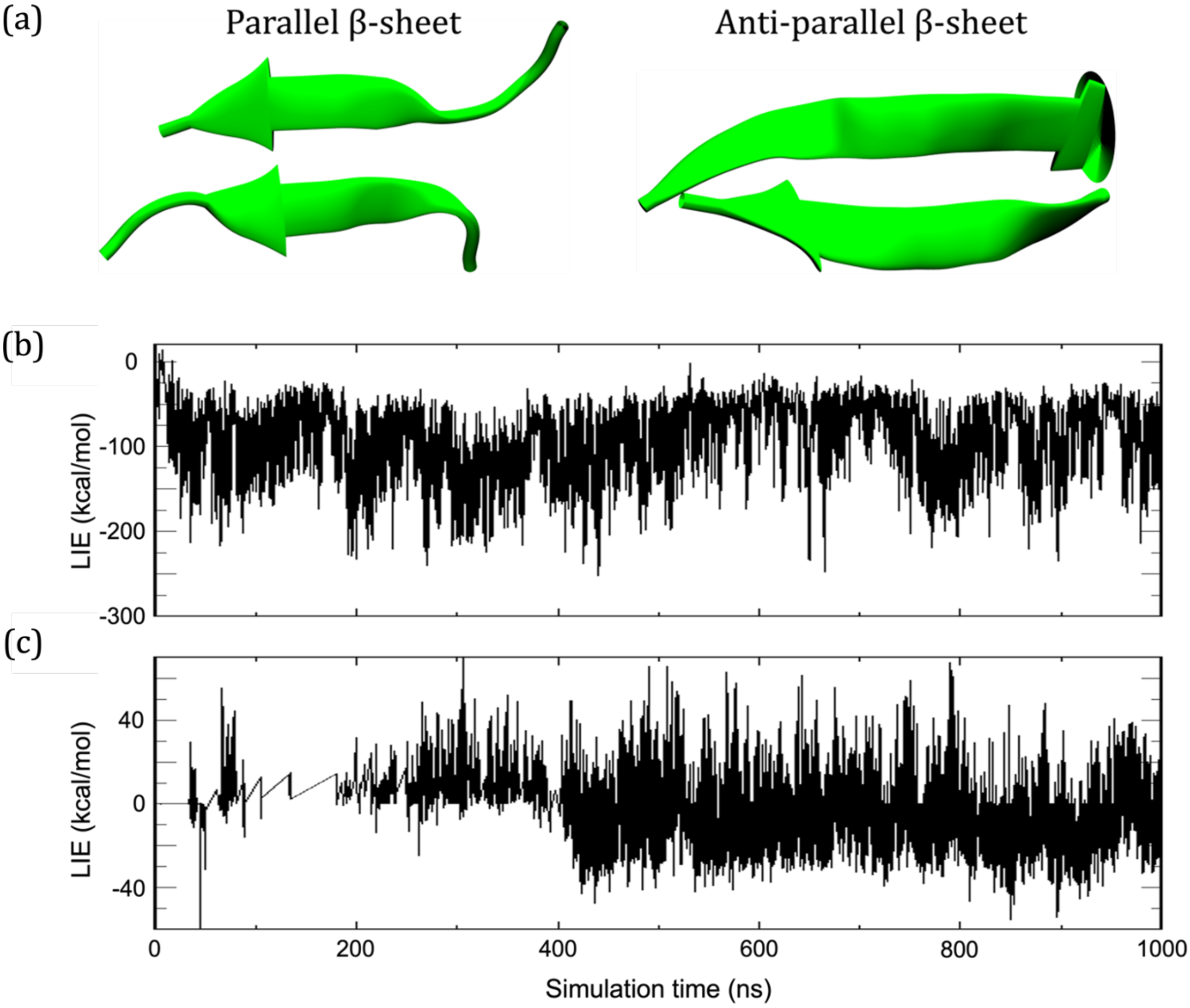

Figure S4: (a) The preferred *β*-sheets under OEEF of strength 0.02 V/Å is parallel, whereas the no-field conditions prefer an antiparallel arrangement. Evolution of linear interaction energies (LIE) between two *β*-strands for the formation of (b) antiparallel and (c) parallel *β*-sheet.

To do so, we first identify the two β-motifs in our MD simulation data, one antiparallel and one parallel, as shown in Figure S4a. Then, we run the MMGBSA calculations from the MD trajectory containing those *β*–sheets and evaluate the energetic differences, which confirms that the antiparallel β-sheet is stabilized by 15.3 kcal/mol relative to the parallel one. Further,

the linear interaction energy plots clarify the energetic differences during the evolution of *β*-sheets from the individual peptide chains (cf. Figure S4b,c). Herein, the energy of the antiparallel *β*-sheet immediately drops with the start of the simulation and begins oscillating around a mean value of approximately -100 kcal/mol (cf. Figure S4b), whereas the parallel one does not show the same trend. Interestingly, the parallel *β*-strands do not even start to interact up to 200 ns and after that, this structure attains a destabilizing positive value until 400 ns (cf. Figure S4c). However, a closer look at the linear interaction energy plots shows that the evolution of the average interaction energy for antiparallel β-sheet (-100 kcal/mol) is way lower than its counterpart parallel one (0 to -10 kcal/mol). To substantiate the result obtained from our MD trajectory, we have further performed the quantum mechanical single point energy calculation and found that the antiparallel β-sheet is stabilized by 69.6 kcal/mol than the parallel one.

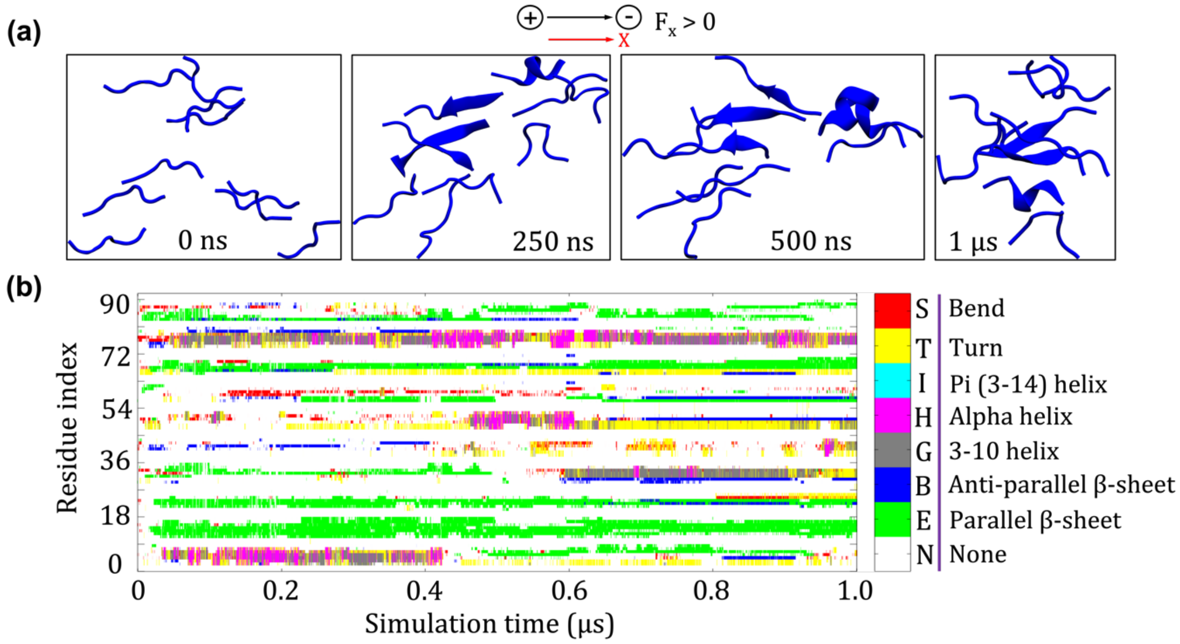

Figure S5: (a) Snapshots showing the behaviour of peptide chains at different time scale in presence of OEEF of strength 0.01 V/Å. (b) Evolution of secondary structure with simulation time. Every 9-residue index signifies a single polypeptide chain.

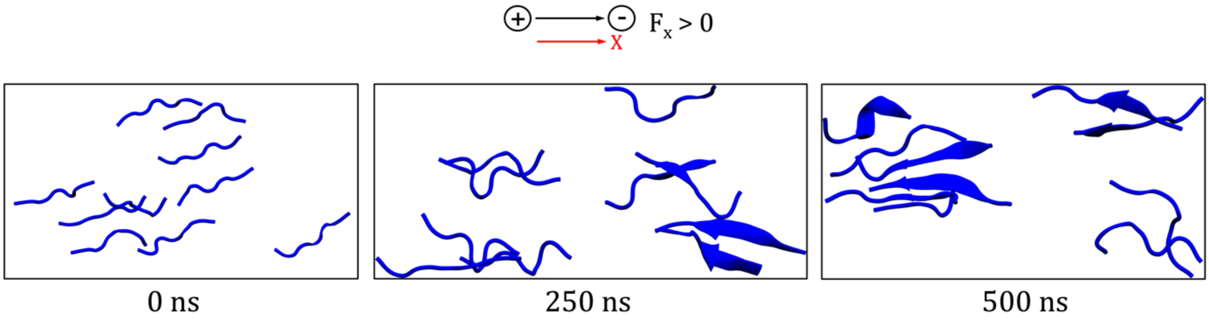

Figure S6: Snapshots showing the behaviour of peptide chains at different time scale in presence of OEEF of strength 0.02 V/Å.

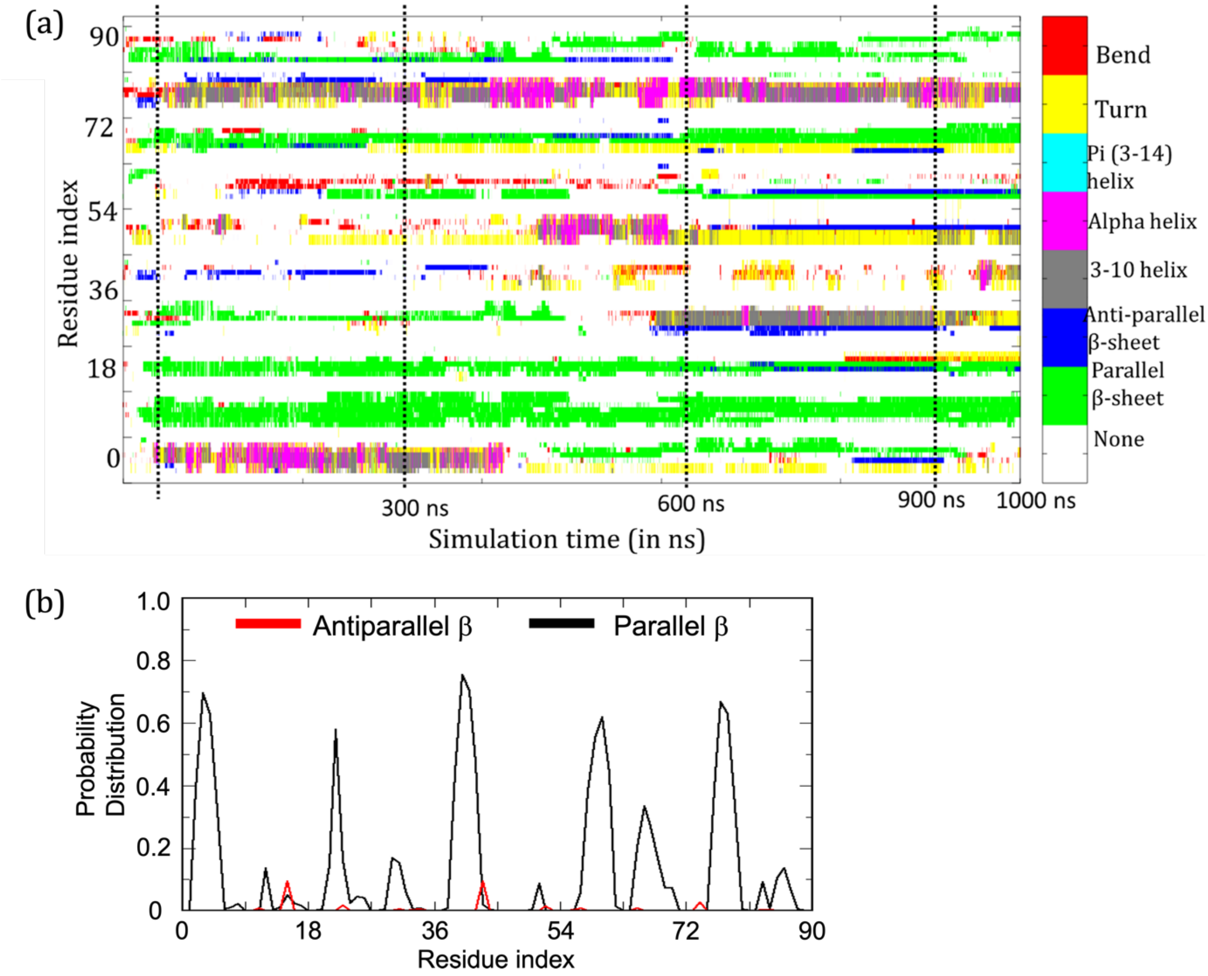

Figure S7: (a) Evolution of secondary structure with simulation time at the OEEF of strength 0.02 V/Å. Every 9-residue index in y-axis signifies a single polypeptide chain. (b) Residue-wise Boltzmann probability distribution plot of parallel and antiparallel β-sheet formation for OEEF = 0.02 V/Å. Every 9-residue index in the x-axis signifies a single polypeptide chain.

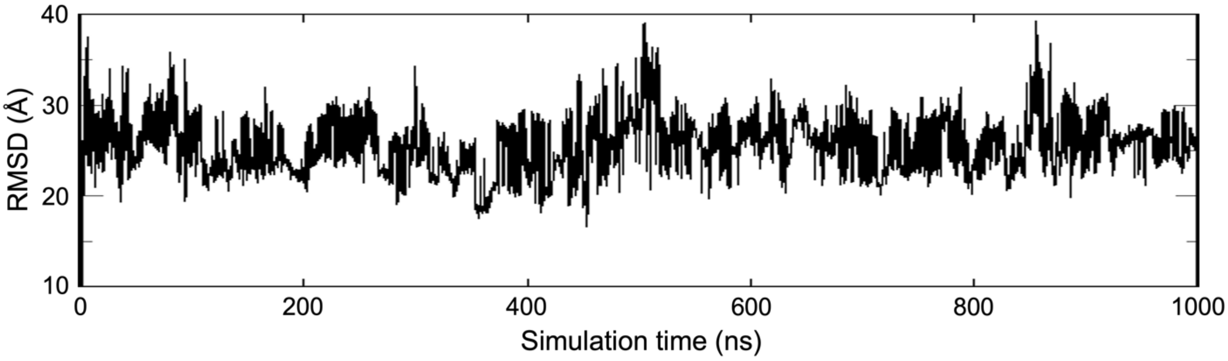

Figure S8: The RMSD plot of the backbone atoms in the presence of OEEF of strength 0.02 V/Å for the entire trajectory with reference to the starting frame.

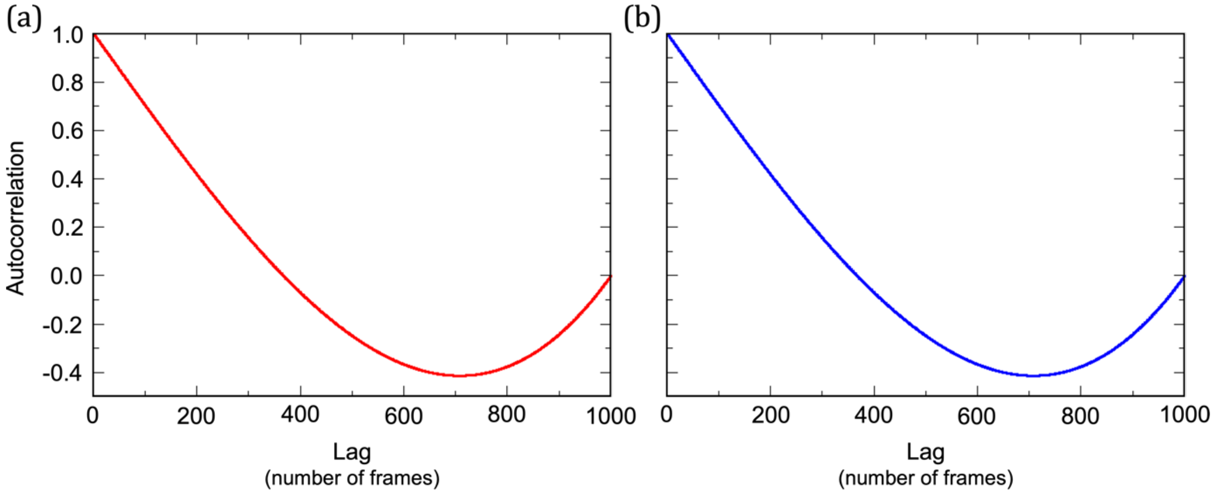

Figure S9: Autocorrelation plot for the evolution of dipole moment with time: (a) in absence of OEEF and (b) in the presence of OEEF of strength 0.02 V/Å. In both cases, we consider the evolution of the dipole moments for the last 50 ns of production MD simulation from their respective trajectories (among 5000 frames, every 5th frame was selected). Note that we obtain an identical pattern of autocorrelation plot for both trajectories, which indicate that the fluctuation of peptides dipole is similar with evolution of time owing to their relative orientation to one another.

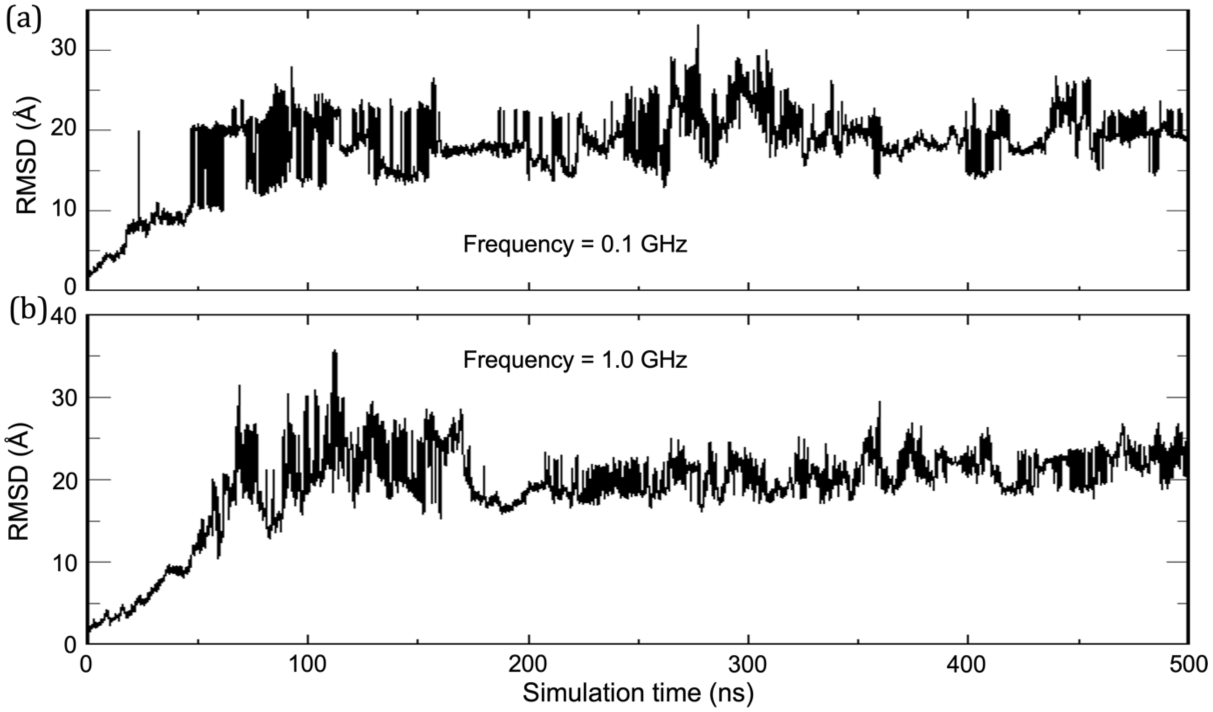

Figure S10: The RMSD plot of the backbone atoms in the presence of oscillating OEEF of strength 0.02 V/Å with a frequency of (a) 0.1GHz and (b) 1.0 GHz for the entire trajectory with reference to the starting frame.

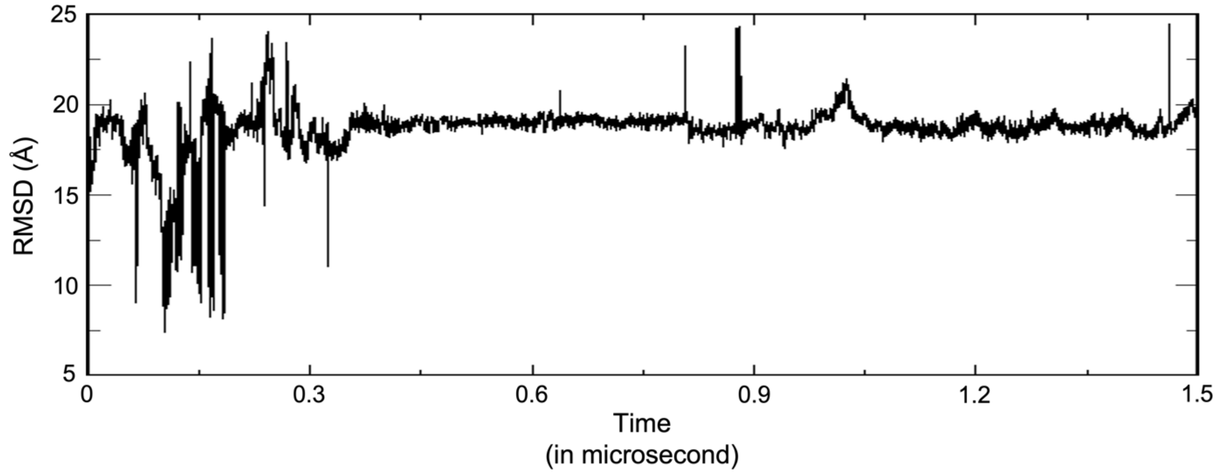

Figure S11: Extended RMSD plot for the simulation which describes the removal of oscillating OEEF of frequency 0.1 GHz (refer to Figure 7).

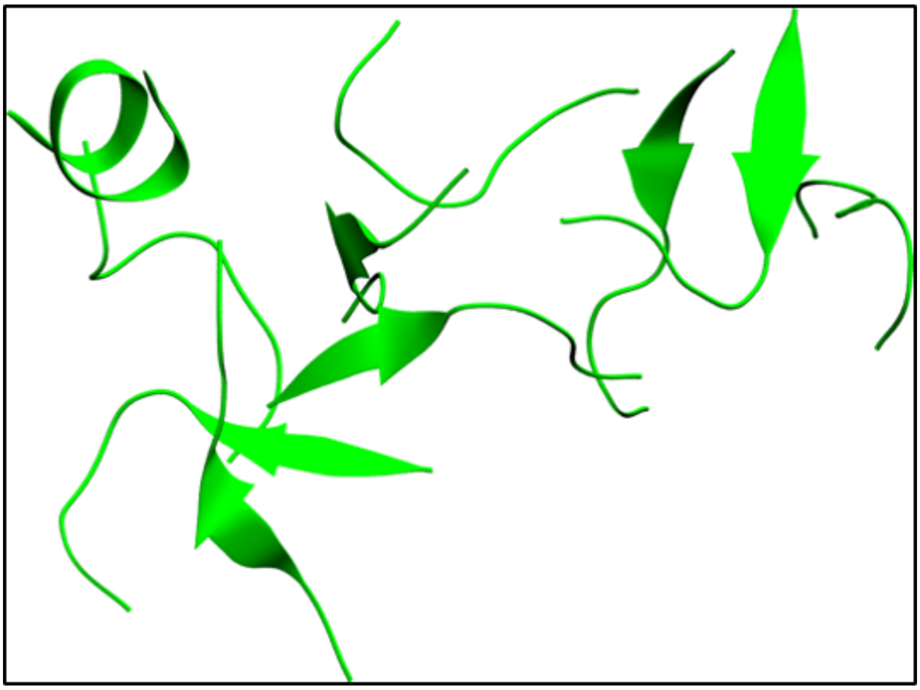

Figure S12: The fate of random neutral peptides as a mixture of half-grown parallel and antiparallel β–sheets after simulation of 1500 ns.
